# The Community of Bilingual English-Spanish Speakers Exploring Issues in Science and Health: Experiences During the COVID-19 Pandemic

**DOI:** 10.15695/jstem/v4i4.05

**Published:** 2021-10-04

**Authors:** Julie E. Lucero, Jenica Finnegan, Joseph Wilcox, David Crowther, Janet Usinger, Ruben K. Dagda, Jacque Ewing-Taylor

**Affiliations:** 1 School of Public Health, University of Nevada, Reno, NV; 2 Latino Research Center, College of Liberal Arts, University of Nevada, Reno, NV; 3 Raggio Research Center for STEM Education, University of Nevada, Reno, NV; 4 Department of Biology, Truckee Meadows Community College, Reno, NV; 5 College of Education and Human Development, University of Nevada, Reno, NV; 6 School of Medicine, University of Nevada, Reno, NV

**Keywords:** Adolescent, COVID-19 pandemic, STEM program, evaluation

## Abstract

Language diversity is increasing in the United States. This growth has implications for language preference, cost, quality, and client outcomes in health services settings. However, language diversity among medical and allied health professionals is lacking. Education pipeline programs are a mechanism to prepare bi- and multi-lingual diverse students to enter health careers. The Community of Bilingual English-Spanish Speakers Exploring Issues in Science and Health (CBESS) is one such program. Through peer mentorship from Leadership Trainees (LT), and a multicomponent 17-month education curriculum, CBESS was designed to increase interest in STEM careers among English-Spanish bilingual high school youth. In 2020, the COVID-19 pandemic interrupted high school students’ education and forced programs to innovate. CBESS was no exception. The most significant modifications were to a) expectations of SRs for a successful Summer Virtual Research Program (SVRP), b) LT roles, and c) scope and delivery of summer science content. A preliminary evaluation was conducted from data collected through pre-post surveys, process data, and focus groups. Among the outcomes were a significant increase in science knowledge among SVRP youth participants as well as no significant differences between cohort 1 and 2 suggesting that changes did not impede program goals. LTs highlighted skills needed and role of mentors. Adaptations were successful and will continue with the 2021 cohort.

## INTRODUCTION

Racial, ethnic, and language diversity continues to grow in the U.S., yet ethnic and linguistic diversity within health careers is not keeping up with current demographics. In the United States, language diversity is at an all-time high with one in five households speaking a language other than English at home (Rumbaut and Massey, 2013; U.S. Census Bureau, 2019). Furthermore, approximately 8% of these households report speaking English less than “very well” (U.S. Census Bureau, 2019). As expected, English proficiency is a strong predictor of English health literacy, or the ability to find, understand, and use health information and services (Jacobson et al., 2016). Thus, health literacy is difficult to achieve for clientele with Spanish language preference. With Latinos as one of the fastest growing ethnic groups (Manuel Krogstad, 2020), there are mounting preferences for Spanish language services and resources. Yet the dearth of bi- and multilingual professionals and resources is unmistakable. The importance of language congruence between service provider-client/patient cannot be overstated.

Cost, quality, and health outcomes are influenced, in part, by language barriers. Service seekers who encounter language barriers have unequal access to healthcare services and more adverse health outcomes compared with patients who do not experience language barriers (Divi et al., 2007; Squires, 2017). For example, the impact of language barriers between patient and healthcare providers results in longer hospital stays, and increased risk of readmission, infection, and medication mismanagement, thereby increasing health disparities (Squires, 2017). The COVID-19 pandemic has worsened health disparities, health care access, and language barriers among Spanish-only speaking Latinos/Latinx which underscores a need for multilingual services, resources, and professionals (CDC, 2020). In the healthcare field, the dearth of bilingual-speaking healthcare providers is abysmal; less than 6% of practicing/licensed physicians are Latinos, even though 27% of the United States population is classified as Latinx (AAMC, 2021). Furthermore, physicians who identify as Latinx are more likely to practice within primary or general care and work in underserved communities (Xierali and Nivet, 2018). An investment in bi- and multilingual students will contribute to a more diverse health workforce.

Despite the growing number of students overall, college-enrolled Latino students still are not representative of the general population. During 2016-2017, 19% of undergraduate students and approximately 11% of graduate students across US institutions identified as Latino (Bauman, 2017). It is well accepted that simply increasing enrollment does not guarantee persistence nor matriculation in healthcare and STEM careers. The COVID-19 pandemic has aggravated these disparities by disrupting finances, academic performance, educational plans, and career goals among undergraduate students of color (Molock and Parchem, 2021). High school students’ education has also been interrupted; COVID-19 has exacerbated education and opportunity gaps (García and Weiss, 2020). The current pandemic has impaired education systems and forced programs to innovate. The purpose of this manuscript is to describe the Community of Bilingual English-Spanish Speakers Exploring Issues in Science and Health (CBESS) program, its successful transition from in-person to online programming for cohort two participants (2020-2021), and how challenges presented by the COVID-19 pandemic were overcome. A preliminary evaluation was conducted by an external evaluator (author 5) to support the assertion of successful programming. An in-depth evaluation of the three CBESS cohorts and respective comparison groups is forthcoming.

### CBESS: An Education Program for Bilingual Youth.

The CBESS program is a cohort-based, multicomponent Science, Technology Engineering, and Math plus health (STEM + health) education program. CBESS is co-led by two principal investigators (authors 6 and 7), coordinated by the second author, and housed within the Raggio Research Center for STEM Education which is directed by the fourth author at the University of Nevada Reno (UNR). CBESS leadership collaborate with inter-collegiate, local and state partners and stakeholders, along with pipeline components of the Nevada IDeA Network of Biomedical Research Excellence (INBRE). CBESS was developed to increase interest in health and STEM careers among English-Spanish bilingual youth in northern Nevada. Three components of the CBESS program are: a) Leadership Trainees (LTs) or near-peer mentors; b) Student Researchers (SRs) who participate in the 17-month multicomponent STEM + health education program; and c) a Community of Practice (CoP) (Wenger et al., 2002), which serves as an advisory committee of local experts. The first author leads the CoP who meet quarterly to stay informed and provide feedback about CBESS programming updates, help to identify potential speakers for the 17-month curriculum, review SR and LT applications, share resources among the CoP network, act as mentors for the community outreach and awareness project, and attend career exploration events, end of summer and 17-month program celebrations.

## METHODS

This section describes the participants, program structure, and programmatic adaptations that were made to the CBESS program because of the COVID-19 pandemic as well as the preliminary evaluation approach.

### Participants.

CBESS began in fall 2017 with funding from the Science Education Partnership Award (SEPA) of the National Institutes of Health. This multi-component education research program utilizes a well-matched intervention-comparison evaluation design. Annually, cohort recruitment begins during the fall months for undergraduate Leadership Trainees (LTs), high school Student Researchers (SRs; intervention group) and the comparison group. Each year five LTs are recruited from across the UNR campus, including the Nevada INBRE biomedical student pipeline program. Eligible LTs are bilingual upper-level undergraduate students interested in healthcare and/or STEM careers. The LT opportunity is posted to CBESS social media platforms, the Latino Research Center bi-weekly newsletter, the CoP network, teacher preparation programs and paper flyers hung in various UNR buildings that house STEM fields (e.g., Colleges of Science and Engineering). Applicant selection is based on interviews, a review of the applicant transcripts and experience, and expressed interest in STEM + health fields. The application review process and participant selection is determined by a committee comprised of CoP members and CBESS leadership team. Promising applicants are invited to an interview where they are asked questions in Spanish. This is an opportunity to gauge bilingual level. Over the 17-month program and under the supervision of the CBESS program coordinator (author 2), the LTs receive extensive professional development training to serve as near-peer mentors SRs and assist the CBESS program coordinator during the 17-month program. LTs are provided a $4,000 incentive for their participation.

Each year, a cohort of up to 32 student researchers (SRs) and 64 comparison students are recruited from various high schools in northern Nevada, predominantly Washoe County school district, and neighboring areas. For example, cohort 1 (C1) enrolled 32 participants while in 2020,28 SRs were enrolled. For each enrolled SR, two well-matched comparison students, based on demographics, are enrolled for a 1:2 intervention:comparison ratio for program evaluation. CBESS applicants who were not selected and students who initiated an application but did not complete it were invited into the comparison group. Students in the comparison group do not participate in any CBESS activities, completed evaluation materials at the same time as intervention students and received a $150 gift card incentive at the end of the 17-month program.

The SRs are predominantly first-generation college goers, and bilingual Spanish-English high school students who have an interest in STEM and/or health careers. Recruitment occurs using multiple strategies including CBESS social media platforms, the CoP network, interaction with high school counselors, and presentations to high school health classes. A promising strategy has been to include a CBESS SR alumni to talk to interested students about CBESS programming and expectations. The program coordinator provides contact information in case an interested student has additional questions. A holistic application process is utilized. Applicants submit demographic and academic information, two letters of recommendation (school counselor, Spanish teacher, and science teacher), three short essays that respond to questions about career interest and experience, and one required essay written in Spanish to gauge bilingual level). Like LTs, the application review process and participant selection are determined by a CoP member and leadership team committee. SRs do not receive an incentive nor are the LTs or SRs required to pay any out-of-pocket fees for their participation in CBESS. Table 1 provides demographics for cohort 1 and 2 intervention students. Evaluation outcomes between intervention and comparison students are forthcoming.

### Program Structure.

An inquiry-based professional development component positions the LTs as STEM insiders and prepares them to engage SRs in STEM-healthcare content as mentors (Fathman and Crowther, 2005; Nasir and de Royston, 2013; Rahm and Moore, 2016). STEM-healthcare LTs receive specialized professional development to prepare them for practicum experiences as CBESS bilingual chaperones, instructor-assistants, and near peer mentors for the SRs. LTs complete training that facilitates the integration of science content, as well as conceptual understanding and contextualization of discipline-specific “academic” language within an inquiry-based “Activity Before Content” (ABC) approach to learning (Crowther et al., 2011; Crowther et al., 2020; Fathman and Crowther, 2005). They also receive instruction in the responsible conduct of research, facilitation and mentoring skills, and learn strategies for positioning linguistically diverse students as “insiders” in STEM-healthcare (see table 2 for summary of LT training). All training is provided by CBESS faculty (authors 1-4), among others and the program coordinator (author 2) supervises all aspects of LT participation.

**Figure 1.**
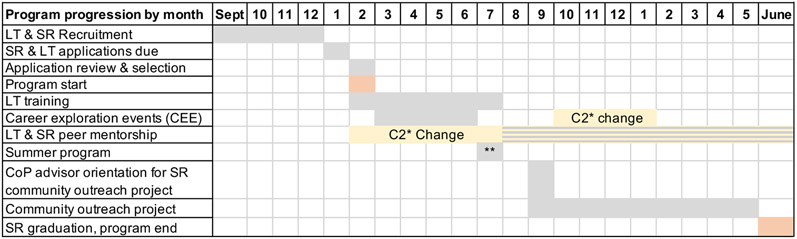
CBESS program’s usual timeline and changes due to the COVID-19 pandemic. *C2 = cohort 2, **The summer program maintained the same timeline but C2 moved to an on-line Zoom format while C1 was in-person and on-campus. The orange boxes indicate the start and stop of the 17-month program. Grey boxes are "usual" timeline, this is what cohort 1 (C1) experienced and yellow boxes indicate changes made to the usual timeline as a result of the COVID-19 pandemic. These changes were experienced by C2. For example, the CEEs were postponed from March until October. Similarly, peer mentorship interactions usually began in August but C2 LT began interactions began nearly 6 months earlier with SRs. The grey and yellow stripes indicate that both C1 and C2 experienced peer mentoring at this time.

The 17-month program for SRs begins in early Spring with 4 monthly career exploration events (see timeline in figure 1). Career Exploration Events (CEE) take place on Saturdays to allow parents to accompany and learn alongside their child. By design, parent engagement was built into the CEE program component as students were required to have a parent or adult family member accompany them to the CEE. Parents had the opportunity to learn alongside their SR. The CEE component was designed to show participants how the variety of traditional and non-traditional disciplines in healthcare and allied health are integrated and allow for engagement with practicing health providers about his/her/their career path. The last CEE also includes a tour of the dormitory in which the students will reside during the summer three-week residential program. The LTs assist in facilitating the CEEs by hosting one of the 4 speaker rooms. Hosting responsibilities include introducing the speakers, leading the question-and-answer portion, and guiding the speakers to their next presentation room. These events last about 3.5 hours, and the LTs are present for approximately 6 hours to assist with setup and cleanup.

During the three weeks on campus, SRs perform hands-on, hypothesis-driven laboratory experiments, take interactive tours of research and anatomical laboratories, research core facilities and clinics, meet with staff from college admissions, financial aid, and first-generation services, and are supported by library services, LTs and CBESS faculty to initiate and complete a youth-led public health research project. The youth-led project culminates with a public dissemination event that provides a valuable opportunity for SRs to present their work to parents, mentors, and CBESS staff. The LTs are responsible for chaperoning the SRs throughout the day and are also required to reside in the residence halls for the three-week summer program. The LTs and SRs arrive on Sunday evenings and check-out on Friday afternoons, having the weekends free.

Following the residential program, SRs, guided by LTs, leverage the result of the youth-led project to develop and carry out a community awareness and outreach project. For example, one group, the Lichen Leaders, sought to understand reasons for anxiety among adolescents. Through their youth-led research project, they conducted a literature review, developed, and administered a survey, and conducted interviews. Their findings suggested that school and social media use were common stressors for teens. To bring more awareness to the issue of anxiety, this group created bilingual informational pamphlets and yard signs promoting positive mental health and well-being. The team distributed 50 pamphlets and posted 20 lawn signs throughout the schools and community locations. Other research topics included lack of blood donors from underrepresented communities, COVID-19 effects on mental health, COVID-19 effects on undocumented immigrants, and distracted and impaired teen driving, among others. LTs meet individually with each of their 5-7 SRs to engage in mentoring sessions which occur monthly through the end of the community awareness and outreach project. The mentoring sessions vary in length, but usually last between 10-20 minutes per student, resulting in a monthly commitment of 1-3 hours.

### COVID-19 Interruptions and Required Modifications.

Due to the COVID-19 pandemic, education systems responded with “emergency eLearning” protocols, marking the rapid transition of face-to-face classes to online learning systems (Murphy, 2020). Between March - May 2020, the UNR campus transitioned to alternative campus operations in response to the COVID-19 pandemic. The University continued to provide online instruction, remote delivery of services and functions, and online, cancelled, or postponed campus events. As expected, CBESS was also impacted. In March 2020, we did not have a clear picture of what was going to happen, if or when in-person programming would be possible. Like most programs across the U.S., when COVID-19 caused by the SARS-CoV-2 viruses emerged in the Spring of 2020, the CBESS staff had to provide the three-week CBESS summer residential program remotely to adhere to prevailing social distancing guidelines at the time. The leadership met and decided to rapidly transition the intensive summer program to a full virtual learning experience via Zoom and to use COVID-19 as the theme for the summer science content sessions. At that point the CBESS Summer Virtual Research Program (SVRP) was born. Additionally, the career exploration events, which were scheduled to occur in Spring 2020, were paused and reinitiated between October 2020 and January 2021. It is worth noting that the COVID-19 pandemic interrupted the conclusion to the community awareness and outreach projects for the first cohort and the residential program for the second cohort. As such, the most significant modifications required were to the a) expectations of SRs needed for a successful SVRP, b) expanded role of the LTs, and c) changes to scope and delivery of summer science content.

### Evaluation Approach.

The evaluation used for both cohort 1 and cohort 2 was based upon an evaluation logic model (not shown) reflective of the inputs, activities, or outputs, and short-, medium-, and long-term impacts. Several methods were used to evaluate the CBESS program and inform necessary changes. First, a summative evaluation of the CBESS program was completed using a pre-post survey for the summer research program. Student researchers and the comparison group were provided the pre-survey to complete at the time that they agreed to participate in the program. The post test was completed immediately after the summer program and at the end of the 17-month program by both the SRs and the comparison group. To assess the comparative impact of the in-person model and the virtual model, the results of the post-assessment survey, administered immediately after the three-week summer research program, were compared.

The survey questions consisted of closed- and open-ended questions, administered electronically through the UNR-licensed Qualtrics system. Closed-ended questions were adapted from the National Science Foundation Scientific Work Experience Programs for Teachers (SWEPT, 2002). The Academic Self-Efficacy survey included nine statements focused on students’ general interest in science and their confidence in succeeding in science classes. A set of 11 statements assessed their self-reported effort toward succeeding in school (academic effort). Eight statements explored students’ knowledge of how to apply to college. Researcher self-identity consisted of five statements to determine the extent to which students understood the typical behaviors of researchers. Assessing student’s knowledge of health careers was determined by asking the extent to which they were able to access and shadow health care professionals. Six statements were used to understand their attitudes about their ethnic identity: four related to being bilingual in their future careers. Finally, six statements focused on their perceptions of the role of CBESS in their futures. To specifically address the changes to the virtual format, questions about knowledge of viruses were added to the survey for cohort 2. (See Appendix A).

To understand the experiences of the LTs, a semi-structured focus group with four of the five cohort 2 LTs (80%) was conducted to get their perceptions on being a mentor and having a larger role delivering the summer content. The semi-structured focus group guide contained the following questions: Describe a way in which CBESS helped you gain skills for your current academic or work status; How were you able to assess student’s knowledge and skills to mentor them throughout this program? and; Would you recommend this LT experience to a friend? Why or why not?

This focus group was conducted using Zoom Video Communication in April 2021. The focus group was audio and video recorded and lasted 60 minutes. The focus group was facilitated by a CBESS intern and MPH student at the University of Nevada. The fifth author and external evaluator observed the focus groups and asked several questions of the participants to further clarify any questions. Having two facilitators who were external to the CBESS program was ethically important to ensure that LTs did not feel coerced to participate or did not censure responses. The focus group was transcribed verbatim and thematically analyzed (Nowell et al., 2017).

## RESULTS

### Program Restructure Due to the COVID-19 Pandemic.

Before implementing the SVRP, CBESS faculty conducted a brief survey to ascertain what concerns SRs had about a virtual curriculum, if SRs were interested in learning more about COVID-19, and how many hours a day they were willing to engage in synchronous activities during the 3-week program. The primary concerns participants expressed were related to access to remote learning technology. For instance, several students either did not have access to a computer or shared a device with other family members. Unstable Wi-Fi signal was also mentioned as a primary concern. In addition, one student was concerned about not having and needing to purchase materials that would be required for at-home experiments. Fortunately, CBESS faculty had previously purchased laptop computers for use by the first cohort and these were readily available to loan to SRs for the duration of the SVRP.

In the first CBESS cohort, the University’s online instruction management system was minimally utilized (Canvas, 2021). In preparation for the substantial shift to online programming, CBESS instructors and program personnel took advantage of the remote-learning platform to scaffold content and events for SR and LT participants including providing access to all presentation materials, access to discussion forums, guest speaker PowerPoint presentations, supplemental handouts, handbooks, Zoom links, assignment tracking, and other materials during the entirety of the SVRP.

Regarding the number of hours per day SRs were willing to engage in synchronous activities, the average response was 3.4 hours, with responses ranging from one to six hours. As most SRs self-identified as Latino, and given that COVID-19 was impacting Latino/Hispanic families in greater numbers (see table 1) - both in terms of mental and physical health and finances compared to other race/ethnic groups (CDC, 2020) - the CBESS team was mindful of competing priorities of SRs and their families. To this end, the CBESS research team decided on providing three hours of daily synchronous activities with additional group meetings, and individual homework as required throughout the three weeks. Eighty percent of SRs reported interest in learning about the health, scientific, and political implications of COVID-19, making it the programmatic theme for the 2020 SVRP.

### Expectations of Student Researchers for a Successful SVRP.

CBESS faculty made all expectations very clear prior to beginning summer programming during “Kick-Off” meetings with each LT / SR group, which helped set up a standard that was maintained throughout the three-week program. This included small details, such as expecting all SRs to log on 10 minutes ahead of start time to ensure that all technology was working properly. If an SR was not logged on by five minutes to start time, their LT would contact them via text or phone call. This ensured that all students were ready to go, and that no time was wasted at the beginning of each daily session. CBESS continues to implement this approach with the current cohort, during CEEs, and will do so again in the next virtual summer program in the 2021/22 cycle. To keep SRs engaged during the SVRP, CBESS team incentivized participation by having weekly raffles for students with university swag as prizes. For instance, students were entered into the raffle each time they asked a question or offered an answer or meaningful comment during synchronous programming. This strategy was not used during the in-person program.

SRs were expected to maintain professional and respectful etiquette throughout the Zoom activities. Most of these expectations were specific to on-line etiquette which is different than in-person etiquette because of the ability of participants to do other things simultaneously without being detected (e.g., mute audio and/or video to take a phone call or watch TV). Because of the physical distance, corrections took longer (e.g., having to text message or direct message a student to stop certain behaviors plus the student would have to see the message). CBESS was also aware that all students would not have a designated desk space, but during orientations, personnel discussed potential strategies with students on how to create a space where they would be able to participate with minimal distractions.

### Expanded Role of the Leadership Trainees.

The LT training occurred as normal (see table 2), albeit through remote Zoom connection. During non-COVID regular programming, SR participants had the opportunity to meet and begin building relationships with LT mentors during CEEs prior to the summer program. Due to the pandemic, all in-person CBESS events scheduled prior to the summer program were cancelled, including the CEEs. The postponement of the CEEs not only expanded the mentoring role of the LTs but also disrupted parent participation as COVID-19 caused competing priorities for parents and CBESS faculty felt they could not require parent participation. As a result, CBESS personnel were tasked with finding opportunities for LTs to develop rapport with SRs and parents in a virtual context. The COVID-19 pandemic and on-line formatting made parent engagement unrealistic.

As a result, in 2020, formal mentoring began prior to the summer program, whereas in the first cohort mentoring began after the completion of the summer program (see figure 1). For the second cohort, the LTs were the primary points of contact for all 29 SRs whereas in the first cohort the program coordinators were the primary source of information. This helped students build rapport with LTs and begin to feel comfortable before summer programming was implemented in July. Furthermore, to maintain a high level of interaction during the pandemic, CBESS faculty also anticipated that SRs would need a contact person to stay up to date with what was going on with CBESS and maintain a connection to the program. For instance, CBESS project coordinators began weekly meetings with LTs to check-in on a personal level and keep LTs updated with project developments.

Additionally, LTs were required to take on several distinct roles during the SVRP, including monitoring and tracking daily attendance and participation of the SRs. In cohort 1, the SRs were living on the UNR campus and attendance was immediately known because the groups lived and traveled together. Whereas cohort 2 SRs were able to log in and out of the Zoom sessions potentially without anyone noticing. Thus, LT involvement in tracking student participation became necessary. The LTs were tasked with privately messaging SRs on the Zoom chat box or via email if SRs were exhibiting any inappropriate or unprofessional behavior such as lying down in bed during the sessions. During the in-person programming the CBESS session faculty (e.g., authors 1,3,4 and/or 5) were expected to address inappropriate behavior. Furthermore, LTs facilitated daily debrief sessions with their respective SR groups. These sessions were scheduled at the group level, sometimes occurring immediately after synchronous programming, whereas some groups were later in the evenings; each group was able to accommodate work and online course class schedules for both LTs and SRs. These debriefs gave SRs an opportunity to connect with their LT and group in a more informal setting without program staff present, allowing for more open discussion and creating a space where students could express their honest concerns or feedback. During in-person programming, LTs debriefed at the end with the SR groups.

For cohort 2, each LT implemented creative ways to engage their students and groups to develop rapport. However, cohort 2 LTs reported that the daily debrief sessions were the most important opportunity for team building and to develop relationships with their SRs. Several LTs had SRs reflect on the day with a “Rose, Thorn, Bud” activity where each SR would identify one highlight or success (rose), one challenge or frustration (thorn), and something they were looking forward to (bud). LTs acknowledged that choosing a different SR to start this activity daily helped each student feel more comfortable and confident in participating. Another strategy that LTs implemented was to play a quick game during debrief sessions that was completely removed from topics related to science or CBESS programming. Examples of games included categories, scavenger hunts and guess the movie. Later in the evening, the CBESS program coordinators would have a separate debrief with all the LTs. Finally, during the SVRP science content LTs were subject to a new training that prepared them to facilitate science modules as described in the next section below.

### Changes to Scope and Delivery of Summer Science Content.

The science content provided during the in-person summer program had previously focused on general aspects of learning science, including biology paired with science and engineering processes (Scientific Methods and Engineering Design Process), or the Nature of Science. Faculty aimed to review some basic concepts and processes in biology (e.g. biochemistry, neuroscience, cell biology), physics and engineering design with students and to demonstrate what these foundational concepts look like in action by providing SRs various hands-on laboratory research experiences in-person within the university environment and within the community. For the most part, while COVID-19 did not change the general idea of the science content objectives, most of the didactic content was adapted to make it relevant to the current pandemic. Content included basic concepts in microbiology (structure of microorganisms, viral structure and transmission, vaccine production, etc.). As such, SRs were able to make connections about how a better understanding of the biology related to COVID-19 could benefit CBESS participants, families, and communities (see table 3).

When creating the learning COVID-19 modules for the CBESS program, authors 3 and 4 decided to approach the concepts from the perspective of a student that had very little to no background in the life sciences. While the students within the summer program may have taken a life science or Biology course prior to acceptance, this approach would allow everyone to be on the same level moving forward with other concepts/modules.

Another consideration was to decide if the modules would be asynchronous or synchronous in delivery. The initial idea was to build asynchronous modules, but after speaking with the students and hearing their opinions about online learning, we decided to build both an asynchronous portion that was followed up with multiple synchronous sessions led by LTs. CBESS collaborated with Educational Innovations, Inc. to build custom science kits with all required materials and supplies. These hands-on materials were shipped in advance to the students directly by Educational Innovations, Inc. so that they could begin the investigations, but then complete them in a synchronous setting.

The COVID-19 trajectory of content learning began with an exploration of the ideas of living (biotic) and non-living (abiotic) elements within our biosphere, moving into DNA as the code of life, expanding into bacteria as the simplest form of life, and finishing with basic viral design and function. This final module led to a more in-depth examination of the coronavirus (SARS-CoV-2, respectively) subgroup. All of these lesson plans utilized the 5E inquiry-based teaching model (Bybee, 2015; Bybee et al., 2006) that included an engagement to introduce the phenomenon, a hands-on activity to explore the phenomenon, an explanation where students watched video clips and did short readings to confirm the content, a second hands-on activity to solidify or elaborate on the idea, and an assessment / evaluative component.

The activities started with an exploration of nature in their neighborhoods to identify biotic/abiotic factors which enabled SRs to think critically about the characteristics of living things. The exploration activity was followed up with another activity that involved the use of jellybeans to illustrate differences in physical characteristics of living and non-living things in classification. The students then learned that within living things, DNA was one of the major criteria of the definition of life. The next module focused on DNA where students created a DNA strand from plastic straw pieces and briefly transcribed it to RNA.

The following module explored bacteria, simply because many people confuse the difference between bacteria and viruses when they become ill (example of getting antibiotics for the flu). The students constructed a Winogradsky column to observe bacteria colonies found in their neighborhoods. To connect bacteria, and eventually COVID-19, to personal health and hygiene, the students then were exposed to the role of handwashing in personal hygiene and as it related to the current emphasis on handwashing due to COVID-19. By using Glogerm™, the students learned proper hand-washing procedures where a hand-held blacklight showed the students where they missed washing their hands thoroughly.

Finally, now that all the basic background was in place, the students learned about viruses in general, how/why they are considered non-living, but contain everything they need to be living, how viruses are classified using the Baltimore Classification System, understanding the role of RNA and mimicking the behaviors of enveloped viruses to reinforce the adaptability and perseverance of COVID-19.

Since the modules were to be facilitated by the LTs, the LTs needed to be trained on both the content and activities so that they could facilitate the synchronous sessions with the SRs. As a reminder, the LTs were upper-level undergraduate students who may not have had any formal or informal educator training. A day was set aside when the creators of the learning modules went through each of the modules with all the LTs and then one LT chose one of the modules to take the lead in presenting to the SRs during the SVRP. Each LT delivered one module during one SVRP day for approximately 20-25 minutes, so this training involved describing the underlying concept of each activity afforded by each kit, performing the activity with SRs (e.g. building the model of the DNA helix), having the LTs reiterate certain points, ask questions to SRs, rehearse occasionally, and clear up any misunderstandings. It also allowed feedback to be given to the authors of the kits if/when there were issues or omissions the LTs discovered. This training was conducted remotely due to COVID-19, but also modeled what some of the learning would look like for the SRs. Every LT seemed comfortable with the material, and their presentation improved with the training.

These modules were presented by CBESS LTs, who setup the topics while providing explanations and the other LTs not instructing provided backup by monitoring the Zoom chat box and responding to questions, while the SRs performed the activities and conducted the write-ups on their own and remotely. The total time was approximately 90 minutes. To further enhance the learning experience afforded by each module, reinforce some basic concepts in microbiology, and relate those concepts to the pandemic current, SRs virtually attended up to 4 different interactive presentations. These presentations were provided by renowned scientists conducting research in basic or applied sciences at the university or state health lab. For example, for cohort 2, in week 1, following the CBESS session on bacteria, a research lecture was given by the Director of Nevada Health State Lab to reinforce lessons learned and to place concepts into practice. During these virtual sessions, the SRs had an opportunity to ask questions to each of the four speakers interactively or on the Zoom chat box. Following each presentation, all unanswered questions from SRs were collected from the chat box and answered by the speaker on a spreadsheet which was provided to SRs the following day. While the subjects broached were far-ranging and deep, these modules were developed in a way to afford accessibility not only with the remote learning delivery but also with the amount and type of information given to the SRs to facilitate conceptual understanding and specialized language that accompanied the content in Spanish and English. Because all the LTs, both program coordinators, and a PI (sixth author) were bilingual they were able to provide explanations, expand on a concept, and/or answer questions in Spanish if that was preferable to students.

## EVALUATION OUTCOMES

### SVRP Participant Outcomes.

The evaluation results presented here reflect the virtual delivery format (cohort 2) between the pre and immediate post, which equals 6 months. In addition, to assess the impact of the virtual model, the results of the post-assessment survey, administered immediately after the three-week summer research program, were compared between cohort 1 (in-person) and cohort 2 (virtual).

Remarkably, CBESS faculty were able to successfully retain 28 SRs throughout the summer virtual research program during the pandemic. Unfortunately, during the second phase of virtual programmatic content, CBESS experienced modest attrition with two students withdrawing during their senior year and the outreach portion of the program; in addition, two SRs completed the post-assessment, but not the pre-assessment. This resulted in the analysis of data from 24 SRs.

An in-depth evaluation pertaining to the effectiveness of the CBESS program on participants and in relation to comparison groups is still ongoing. However, in brief, the summative evaluation data show the summer virtual residential program (SVRP) was successful as suggested by the evaluation data. For instance, summative evaluation data demonstrated that SRs improved with respect to “Self Confidence in Knowledge of Science” (mean 2.00 pretreatment vs. 1.89 post-treatment, N=24 students, paired t-test) while no significant differences or positive trends regarding bilingualism as an asset and CBESS-related factors were observed. In addition, (>60%) of SRs strongly agreed that CBESS provided the necessary knowledge and tools to apply to college and for pursuing STEM and health careers (data not shown). Interestingly, the comparison group (N=37), which did not receive treatment, performed significantly worse in some areas, including bilingualism, ethnicity, and attitude towards college, suggesting that students that did not receive the educational content worsened in terms of their attitude and insight towards being bilingualism as an asset and ethnicity, presumably due to the anxiety, uncertainty and mental stress caused by the COVID19 pandemic during the summer and fall of 2020. In contrast, the SRs benefited from substantial near peer and adult mentoring to engage and follow up on their well-being.

From the process evaluation, there was overwhelming agreement among the 24 SRs that the program was well planned and implemented. When asked to rate their agreement with the statement, “*the expectations for the program were clear*,” 85% *strongly agreed* and 15% *agreed* with the statement. When asked to rate their agreement with the statement, “*I knew who to go to with questions and how to find her/him*” 85% *strongly agreed*, 11% *agreed*, and 4% selected *neutral*. When asked to rate their agreement with the statement, “*I understood what I was supposed to present and how*,” 78% *strongly agreed*, 15% *agreed*, and 7% were *neutral*. Furthermore, when asked about skills learned during the program, SR participants responded “Research, collaborative, and communication skills” as well as “Researching in different databases and writing in APA format.”

In addition to being asked to rate the program, SRs were asked the open-ended question: *Do you have any general-thoughts or comments about the CBESS Summer Virtual Research Program or suggestions to improve future events?* Six SRs expounded on how great the summer program was, with no reference to the format. Responses included suggestions for ice breakers, more labs and experiments, workshops on making graphs and charts. One mentioned the “amazing” mentors because “they were what truly made my day” while another stated that it was the “best experience” of the summer. Six SRs reported that it was a great experience, but references were made to the program being virtual. A typical response in this group was, “Although the summer program was all virtual, I enjoyed the entire three weeks, with the science kits and meeting new organizations, it was definitely an experience.” However, it must be noted that two SRs specifically expressed a desire to get to know other SRs in person and three SRs were more emphatic about their desire to have the program in-person. One stated, “I think the virtual part lost the personal connections or relationships that could have happened.” Perhaps typical of suggestions for improvement, four indicated a desire for more time. Two SRs mentioned the research project and other assignments; another wanted longer breaks; another wanted more discussion about college options.

### Comparison of Participant Outcomes.

To understand if the changes in 2020 impaired program goals, independent sample *t*-tests were conducted on each set of scales. No significance differences were found between the two groups (see table 4). The lack of significant difference is a good indicator that the programmatic changes did not impair the programmatic goals for cohort 2 SRs.

### Expanded Role of the Leadership Trainees.

From the qualitative data two themes emerged a) Mentorship skills, b) Mentorship role. These themes are discussed next and selected quotes are presented as supporting evidence.

#### Mentorship Skills.

Leadership trainees reported that they learned how to work with a diverse group of students. Specifically, the LTs gained skill needed for mentoring, such as active listening. For example, one participant stated, “I think really one of my number one skills is having patience and really learning how to listen, because I think it’s easy to put yourself in a role, you know you’re in college these kids are looking up to you.” Additionally, the expanded role of the LTs in cohort 2 required time management, a skill that was reinforced during the summer program. As another LT stated,

The students learn that but I learned that as well, and time management and all those things but not just school but in everything else that we had to do during CBESS and that was a great skill to learn as well and It just helped me realize how important everyone in the Community is and how important research is.

#### Mentorship Role.

LTs expressed that being in a mentorship role came with the need to set boundaries and regular communication. The leadership trainees reported having good relationships with their students that allowed them give information and advice for the work they were doing, but setting boundaries was necessary. For example, one LT stated,

Being able to kind of have that bond with your students but also being able to set boundaries has really kind of highlighted the importance of being able to specify certain professional positions in your life right, so I think that the CBESS program has definitely highlighted how to be able to interact with other people.

To build a solid foundation for a mentor-mentee relationship, leadership trainees regularly interacted with SRs through monthly check-in meetings. Over the course of the program, career paths became a common discussion point and the more the SRs learned, their interests diversified, “Having those monthly meetings and we talked with them about what careers they’re thinking of now, like the evolution of it [career selection] and was nice to kind of see their growth.” All in all, the LTs expressed the personal benefit of mentorship; the pride that comes from being a part of and witnessing student growth. For example,

I think that I’ve seen a huge change within our students and with that being said, I feel some type of pride almost being able to realize that we’ve come so far within the program and realizing that not only has there been a personal impact within us as mentors, but also within our mentees because they’ve done a lot.

#### Changes to Scope and Delivery of Summer Science Content.

As mentioned in the Methods section of this manuscript, while most of the science content was significantly modified in response to the pandemic, the basic concepts and learning objectives were not. To make the content relevant to the current pandemic and prepare/equip SRs to undertake the youth-led community health project, SRs were exposed to basic concepts in cell biology and microbiology as well as inculcating basic techniques in data collection, preparing/administering surveys, data analysis, data presentation and dissemination of results. In aggregate, the summative evaluation pre/post data showed that SRs gained significant knowledge in some basic concepts in microbiology and cell biology (items 1, 2, 5, 7, 10, 11 and 13) whereas SRs did not perform better or worse in other concepts. However, in aggregate, SRs performed better in all 15 questions administered to them with a positive overall mean of +14.77% (Table 5).

## CONCLUSION AND FUTURE DIRECTION

Despite the current pandemic disrupting social life for SRs and in-person instruction, our summative evaluation data showed that the second cohort of SRs recruited by CBESS had an overall positive experience during the virtual SVRP. Indeed, our evaluation data showed significant improvements in attaining basic concepts in biology (microbiology and cell biology), enhanced interest in STEM careers, and on the importance of leveraging their bilingualism as an asset by which bilingual speakers can enhance their learning of STEM content through a different didactic lens. In the next phase of CBESS, a hybrid program is planned to represent strengths and qualities of in-person and remote-learning modes. Decision on mode of interactions will be built based on qualitative and quantitative summative evaluation data (forthcoming).

It is worth noting that student learning and engagement were not only retained for cohort 2 but some significant gains in student development (social aspects, bilingualism and enhanced interest in careers) were enhanced similar to cohort 1 in 2019 suggesting that CBESS was able to successfully provide an overall positive experience during the pandemic. Importantly, our evaluation data showed that CBESS in-person programming is adaptable to long distance learning, synchronous/dynamic activities can be maintained by shipping science kits to student, can be scaled and scaffolded with science learning modules, and is resilient in response to social/environmental crisis such as the current pandemic. Through LT focus group participation, it became apparent that listening and time management skills are essential for being a mentor. However, while many aspects of student learning, CBESS programming, and mentorship were retained for cohort 2, we recognize that parents of SRs were not engaged significantly as in cohort 1 which predominantly occurred during the CEEs.

CBESS faculty realized that this was a flaw in the program design. As part of ongoing efforts to sustain CBESS, we are looking to engage high school counselors and administrators from both urban and rural high schools in northern Nevada to support a learning community to provide STEM/ health bilingual resources and parent engagement. Secondly, we will have LTs meet with parents or caregivers in addition to SRs during program check-ins. Going forward, CBESS will not only strive to provide instruction and mentorship remotely for cohort 3 and beyond but will add parent engagement activities that invite parents to Community of Practice events, parent-instructor focus groups, CEEs and youth-led dissemination events.

Evaluation data reported in this manuscript have focused on only the changes to programming that were necessary because of the current pandemic. With the third cohort completing the program in 2022, a comprehensive evaluation is currently underway. Future evaluation reports will detail program outcomes for three cohorts of intervention and comparison students. Qualitative and quantitative, pre and post program data, received from three cohorts of leadership trainees is also under review. However, we have noted that the data obtained from focus groups are more meaningful and relevant, especially given the small number of LTs for each cohort (4-5 people). A 5-year longitudinal assessment of career interests and paths chosen by SRs and LTs from cohorts 1-3 is also on-going. These data are preliminary and will be included in follow-up reports as CBESS cohort 1 students, in 2021, will be third-year program alumni.

## Supplementary Material

Appendix A

## Figures and Tables

**Table 1.** Aggregate Student Researcher Demographics, Cohort 1 and 2.

Demographic Category	Cohort (1 %)	Cohort 2 (%)
sex		
Female	23 (72%)	24 (86%)
Male	9 (28%)	4 (14%)
Race/ethnicity		
Hispanic / Latino	30 (94%)	27 (96%)
Asian	1 (<1%)	1 (4%)
More than 1 race	1 (<1%)	
First-generation student	32 (100%)	28 (100%)
Geography of school		
Urban	27 (84%)	26 (93%)
Rural	5 (16%)	2 (7%)

**Table 2.** Topics, description, and length of training provided to Leadership Trainees.

Training Topic	Facilitator	Length	Description
STEM Identity	Dr. Lynda Wiest, CBESS Faculty	4 Hours	In this training, LTs examine foundational concepts regarding underrepresented groups in medical careers, discuss the role of identify in relation to STEM, and articulate issues and challenges underrepresented groups might face in post-secondary contexts. LTs are then challenged to discuss strategies that can be implemented to help high school mentees be self-directed in their education pursuits.
Inquiry-Based Instruction	Dr. Dave Crowther, CBESS Co-I Dr. Joey Wilcox, Faculty	2 hours	Facilitators review the nature of science, and pedagogical / instructional shifts in K12 science education. The three dimensions (Disciplinary Core Ideas, Science and Engineering Practices, and Cross Cutting Concepts) are discussed as a framework for Next Generation Science Standards.
STEM Modules	Dr. Dave Crowther, CBESS Co-I Dr. Joey Wilcox, Faculty	3 hours	Module creators assist LTs in organizing their at-home science kits and then practice each hands-on activity. The PowerPoints are reviewed, and participants engage in open discussion about how the modules can be implemented.
Literature Reviews	LT / Writing and Speaking Center	1 Hour	CBESS was lucky to have an LT who was also employed by the University Writing and Speaking Center. This LT was able to lend her expertise and experience with literature reviews to train both the other LTs and the SRs in conducting them. The training including developing a research question, the objectives of a literature review, and conducting database searches.
Facilitation	Dr. Julie Lucero, CBESS Co-I	1 Hour	This training included understanding the role and characteristics of a facilitator. Specific facilitation behaviors such as setting ground rules. Garnering participation and resolving conflict are discussed.
Responsible Conduct of Research	CITI / Research Integrity Office	CITI=~5 hours RIO= 1 hour	All LTs complete the Collaborative Institutional Training Initiative (CITI Program) for Social and Behavioral Research. Additionally, the University Research Integrity Office has also offered trainings that included an overview of the IRB, history of and examples of unethical research, and an introduction to IRBnet.
Mentoring	Noemi Gomez Martinez, Jenica Finnegan, CBESS Project Coordinators	4	Training topics included information regarding mandatory reporting, developing rapport, logistics for mentoring sessions and logging contact notes, self-care, maintaining boundaries and the role of a mentor.

**Table 3.** Summer CBESS program. Week 1 daily topics to illustrate the differences between the in-person and virtual programs.

Week cohort, year, and modality	Monday	Tuesday	Wednesday	Thursday	Friday
Cohort 1 2019 In-person	Nature of Science and Scientific Methods	Engineering and Design	Researchable Questions	Social Determinants of Health, Socioecological Model	Understanding DNA structure/function & Cheek Extraction
Cohort 2 2020 Virtual	Living / Non-living - What is life?	Understanding DNA structure/function Extract plant DNA	Bacteria	Viruses	Social Determinants of Health, Socioecological Model

**Table 4.** T-test comparison of cohort 1 and 2 intervention group: post assessment.

Concepts	Cohort 1 (N=32)	Cohort 2 (N=24)	P-value
Academic self-efficacy	1.66	1.71	0.62
Academic effort	1.55	1.67	0.32
College readiness knowledge	2.71	2.72	0.98
Researcher self-identity	1.29	1.50	0.08
Access to health care professionals	2.11	2.37	0.15
Sense of ethnicity	1.54	1.40	0.43
Perceptions of being bilingual	1.65	1.54	0.54
Role of CBESS in future career	1.32	1.53	0.07

**Table 5.** Science knowledge: correct percentage per question (N=27)

(A)biotic, DNA, Bacteria, and Virus Knowledge Assessment	Pre- test	Post- test	% change
1. For something to be considered “living” it can eliminate solid and liquid waste	48%	78%	+30
2. The sun is the ultimate source of energy for almost all organisms on planet Earth	81%	89%	+8
3. Grasses and dandelions are organisms that move materials from the abiotic components of an ecosystem into the biotic components, and by photosynthesis.	30%	30%	0
4. The correct sequence in the taxonomic hierarchy	26%	67%	+41
5. A membrane bound nucleus is the major difference between a Prokaryotic and a Eukaryotic cell	48%	81%	+33
6. DNA stand for Deoxyribonucliec Acid	85%	85%	+1
7. DNA become RNA through transcription	59%	70%	+11
8. Protein Central is the process when DNA becomes RNA and then a Protein	52%	81%	+29
9. When a bacterium can live without oxygen it is anaerobic	67%	89%	+22
10. Archaebacteria are bacteria that live in extreme environments	70%	100%	+30
11. Prokaryotes are cells/organisms without a membrane bound nucleus	59%	81%	+21
12. There are five classifications of viruses	48%	33%	−15
13. Lysing is the process of a virus exiting a cell	48%	63%	+15
14. Knowledge of when viruses need a cell	93%	85%	−8
15. Sharing a soda with an infected person is an example of an action that spread the COVID 19 virus to others	93%	96%	+3

## ABBREVIATIONS

ABC: “Activity Before Content;”
CBESS: Community of Bilingual English-Spanish Speakers Exploring Issues in Science and Health
CEE: Career Exploration Events
CoP: Community of Practice
INBRE: IDeA Network of Biomedical Research Excellence
LT: Leadership Trainees
SEPA: Science Education Partnership Award
SR: Student Researchers
SVRP: Summer Virtual Research Program
SWEPT: Scientific Work Experience Programs for Teachers
UNR: University of Nevada Reno
