# Supplementary material for: The Community of Bilingual English-Spanish Speakers Exploring Issues in Science and Health: Experiences During the COVID-19 Pandemic
Source: J STEM Outreach. Author manuscript; Available in PMC 2022 Oct 4. (PMC8653503; doi:10.15695/jstem/v4i4.05)
Supplement: Appendix A [file NIHMS1751163-supplement-Appendix_A.docx]

**Appendix A**

Academic Self-Efficacy

1. Science classes can help prepare me for a career in science or health.
2. Science is interesting to me.
3. I am enthusiastic about science.
4. I am interested in science or health as a career.
5. I think that a science or health career is possible for me.
6. I know I can be successful in a STEM-healthcare career.
7. I am confident that I can get good grades in a biology class.
8. I am knowledgeable about science.
9. I have a good understanding of what science is all about.

Academic Effort

1. I am certain I can master the skills taught in my classes.
2. I am certain I can figure out how to do the most difficult work in my classes.
3. I can do almost all the work in my classes if I did not give up.
4. Even if the work in my classes was hard, I can learn it.
5. I can do even the hardest work in my classes if I try.
6. I try hard to do well in school.
7. In class, I work as hard as I could.
8. When I am in class, I participate in class discussions.
9. I pay attention in class.
10. When I am in class, I feel good.
11. When we work on something in class, I feel interested.

College Readiness - Knowledge

1. I know the deadlines for the various steps in the college admission process.
2. I know how to select colleges that are likely to accept me.
3. I understand the difference between a community college and a four-year college.
4. I know where to find information on the colleges I am interested in attending.
5. I know how to complete a college application.
6. I know who to ask for college recommendation letters.
7. I know how to write an essay for college applications.
8. I know how to apply for financial aid from colleges.

Researcher Self-Identity

1. I know how to locate reliable health information on the internet or in the library.
2. I know how to write a scientific question.
3. I know how to use Excel, PowerPoint, or another computer program to create graphs and tables for displaying research results.
4. I know how to create a scientific poster presentation.
5. I know how to give a scientific research presentation.

Access to Health Care Professionals

1. I have shadowed (followed) a health career professional, such as a doctor, nurse or medical technician.
2. I have talked to a health career professional about his/her career.
3. I know about many different health careers.
4. I can approach a health career professional for information about health careers.

Sense of Ethnicity

1. I have a strong sense of belonging to my own ethnic group.
2. I understand pretty well what my ethnic group membership means to me.
3. I have often done things that will help me understand my ethnic background better.
4. I have often talked to other people in order to learn more about my ethnic group.
5. I feel a strong attachment towards my own ethnic group.
6. I have spent time trying to find out more about my ethnic group, such as its history, traditions, and customs.

Perceptions of Being Bilingual

1. Knowing a second language will help me get accepted to college/certification programs or get hired for a job.
2. Having English as my second language is an asset (answer only if English is your second language).
3. Being Spanish-English Bilingual will make it possible for me to enter a STEM-healthcare career.
4. It is difficult for ethnic minorities to succeed in science and health careers.

Role of CBESS in My Future

1. Participating in CBESS will prepare me for applying to colleges.
2. I know I can be successful in a STEM-healthcare major in college.
3. Participating in CBESS will help prepare me for a career in STEM-healthcare.
4. I have made personal connections with people during my CBESS experience that are willing to mentor me in the future.
5. I have made personal connections with people during my CBESS experience that will help me get into college or certificate programs.
6. I have made personal connections with people during my CBESS experiences that will help me get a job.
